# Diagnosis and Treatment of Ectopic Pregnancy in a Cesarean Section Scar—Case Report

**DOI:** 10.3390/jcm15062302

**Published:** 2026-03-17

**Authors:** Polina V. Kulabukhova, Tatyana V. Fokina, Maria N. Babaeva, Aleksandra V. Asaturova, Natalia V. Nizyaeva

**Affiliations:** 1 Federal State Budgetary Institution “National Medical Research Center of Obstetrics, Gynecology and Perinatology Named After Academician V.I. Kulakov” Ministry of Health of the Russian Federation, 117997 Moscow, Russia; kulpola@mail.ru (P.V.K.); a.asaturova@gmail.com (A.V.A.); 2 Avtsyn Research Institute of Human Morphology of Federal State Budget Scientific Institution, Petrovsky National Research Center of Surgery, 119435 Moscow, Russia; tatyana-doc-6@mail.ru (T.V.F.); aneira@mail.ru (M.N.B.)

**Keywords:** ectopic pregnancy within the scar, scar after cesarean section, chorionic villus invasion, magnetic resonance imaging

## Abstract

**Background/Objectives:** Post-cesarean section scar niche pregnancy is one of the rarest forms. It is characterized by implantation of the gestation sac within the scar niche and is often associated with chorionic villi adhesion into the thinned cesarean section scar. The increasing incidence of this condition is associated with the increasing frequency of cesarean sections and the widespread use of ultrasound in early pregnancy. The most significant clinical findings are the detection of chorionic villus invasion and uterine wall insufficiency, which may be detected using magnetic resonance imaging, including contrast, and are crucial for determining patient management. This pathology may be considered life-threatening due to complications such as early uterine rupture with bleeding, which, if not diagnosed promptly, can lead to hysterectomy and loss of the woman’s reproductive health. Early diagnosis allows for the use of conservative treatment methods, preserving the uterus. The aim of the study is to clarify the clinical practices to follow in cases where an MRI examination with contrast agent is indicated to be performed on a pregnant patient. **Methods:** Ultrasound and MRI examination with counter-rotation, as well as histological and immunohistochemical examination of the remnants of the gestational sac were performed. **Results:** A 36-year-old pregnant woman was hospitalized in her eighth week of pregnancy with complaints of vaginal bleeding and persistent abdominal pain. An ultrasound scan revealed a pregnancy of 8 weeks and 5 days, and a low-lying chorion in the isthmus of the uterus, along with thinning of the cesarean scar and the formation of a scar niche resembling a hernia. Early signs of chorionic invasion were not treated. An MRI revealed signs of superficial chorionic adhesion to the cesarean scar, both to the isthmus and the internal os. Given that the woman did not wish to continue the pregnancy, uterine artery embolization was performed to reduce potential blood loss. Subsequently, laparoscopy, adhesiolysis, vacuum aspiration of the gestational sac, uterine curettage, hysteroresectoscopy, and coagulation of the fetal bed were performed. Histological and immunohistochemical examination revealed signs of inflammation in the area of the suspected lesion. **Conclusions:** This case report shows the potential value of MRI in complex cases of ultrasound detection of a gestational sac within scar tissue. MRI was used to assess the location of the gestational sac and evaluate the thickness of the cesarean scar to detect its dysfunction. Furthermore, contrast enhancement of the MRI may be useful in the most complex cases but requires an informed consent discussion with the patient. However, the latter issue requires discussion and proof of its safety for the fetus.

## 1. Introduction

The increasing number of women delivering by cesarean section has led to the problem of ectopic pregnancy in the scar after cesarean section (CS), and may cause life-threatening complications such as bleeding and uterine rupture [1]. In the case of attachment of the gestational sac (GS) in this area, it can lead to excessive placental invasion into the uterine wall or placenta accreta spectrum disorder (PAS) [1,2,3]. Along with this, the incidence of PAS increases from 1.7 per 10,000 births overall to 577 per 10,000 births in women with both a previous caesarean delivery and placenta previa [1 JC]. The mortality rate in pregnancy in the scar is 191.2 per 100,000 cases, which is 12 times higher than the mortality rate in tubal pregnancy [2]. Risk factors for this pathology include two or more cesarean sections, endometritis in the postpartum period, curettage of the uterine cavity (including abortion), spontaneous miscarriages, chorion previa, and a short interval between the onset of pregnancy and the previous CS [3,4].

However, particular importance is attributed to the weakness and thinning of the scar following a previous cesarean section, as well as the presence of immature scar tissue with pathological vascularization [5].

Considering the dangerous complications, patients often undergo a hysterectomy, leading to further infertility [6].

The primary diagnostic method is ultrasound, which, as early as 8 weeks, can reveal visual signs gestational or fetal sac, thinning of the anterior uterine wall and the cesarean section scar (down to 1–3 mm) and others [7].

Although there are various classifications for assessing the location of the gestational sac in relation to the scar niche, the sonographic system for assessing pregnancy in the scar illustrated [8] describes a new grading system for pregnancy in the scar niche pregnancy (SNP). This system divides scar pregnancy into four categories, depending on the depth of implantation of the gestational sac in scar tissue after a cesarean section. Grades III and IV are more dangerous and associated to more aggressive treatment, such as hysterectomy.

- Grade I—the gestational sac (chorion) is implanted into less than half of the myometrium.
- Grade II—the gestational sac extends into more than half of the myometrium.
- Grade III—the gestational sac extends beyond the myometrium and serosal membrane of the uterus.
- Grade IV—the gestational sac becomes an amorphous tumor with abundant vascularization in the scar after a cesarean section.
- However, in complex and controversial cases, MRI is used. MRI is a complementary diagnostic method to assess parameters such as the location of the gestational sac and signs of thinning of the lower uterine segment. It is very important to visualize the degree of chorionic invasion into the uterine wall and uterine isthmus. Its most valuable feature is the ability to determine the depth of chorionic invasion, including involvement of adjacent organs.
- According to the literature, SNP may be divided also into two types. Type 1 (endogenous) is where the initially implanted gestational sac in the cesarean scar gradually invades the uterine cavity [9]. In this case, there is a chance to prolong the pregnancy until the third trimester and give birth to a viable child. Type 2 (exogenous) occurs when the gestational sac is implanted in the cesarean scar, penetrating deeper towards the serosal uterine surface [9].
- Based on MRI analysis, it is possible to classify the location of the gestational sac relative to the scar niche, which directly impacts the pregnancy outcome and prognosis. SNP may be divided into two [9] or three types [9,10,11,12]. In accordance with the classification, exogenous type [9] or type III [12] carry a higher risk of massive bleeding and uterine rupture in the first trimester of pregnancy [10,11,12].
- T2-weighted images (T2WIs) in the sagittal plane are used for assessment. The location of the gestational sac is the determining factor. Therefore, Type II is characterized by its localization partially within the scar niche and partially within the uterine cavity. This type of pregnancy is not always associated with the risk of scar rupture and bleeding as the pregnancy progresses, as the gestational sac is partially located within the uterine cavity. This type of pregnancy can be dynamically monitored and prolonged at the patient’s insistent desire. However, as pregnancy progresses, placenta accreta and placenta increta of varying severity may occur. But, in cases of late diagnosis, massive bleeding, and the woman’s unwillingness to preserve her reproductive health, patients undergo total hysterectomy [11,13]. With timely diagnosis in the early stages of gestation, the patient can undergo treatment, including hysteroresectoscopy with removal of the gestational sac, or use more gentle methods or try prolong the pregnancy. In cases of CS damage and its failure, one-stage metroplasty can be performed, which allows preservation of fertility. To reduce the blood supply to the pelvic organs, uterine artery embolization may be performed beforehand [14,15].
- Unfortunately, according to current clinical guidelines, the use of contrast (for both ultrasound and MRI) is limited in pregnancy, which prevents its widespread use in clinical practice. Moreover, it would be crucial for practicing physicians to understand how contrast enhancement improves diagnostics. In the presented case, given that the woman did not wish to continue her current pregnancy, it would have been possible to use contrast enhancement during MRI and demonstrate its potential for clinical practice [16].
- The aim of the study is to clarify the clinical practices to follow in cases where an MRI examination with contrast agent is indicated to be performed on a pregnant patient.

## 2. Case Report

A 36-year-old female patient was admitted in September 2022 to the Operative Gynecology Department of the V.I. Kulakov National Medical Research Center of Obstetrics, Gynecology and Perinatal Gynecology. She presented with complaints of bloody vaginal discharge and abdominal pain. The patient had a history of cesarean section in 2019, as well as two missed abortion pregnancies with curettage in 2021 and 2023. This pregnancy was conceived spontaneously. Ultrasound examination revealed a progressing pregnancy of 8 weeks and 5 days’ gestation. Early signs of chorionic invasion could not be ruled out.

**Figure 1:**
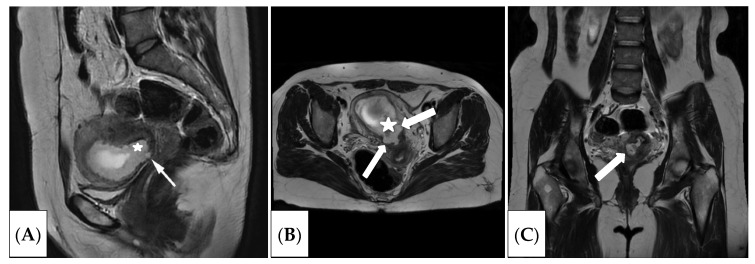
MRI T2WIs in the sagittal (**A**), axial (**B**), and coronal (**C**) planes showing the gestational sac within the uterine cavity. The chorion was identified in the isthmus of the uterus (indicated by an asterisk), with fragments extending into the scar from the caesarean section and the isthmus of the uterus (indicated by a white arrow).

**Figure 2:**
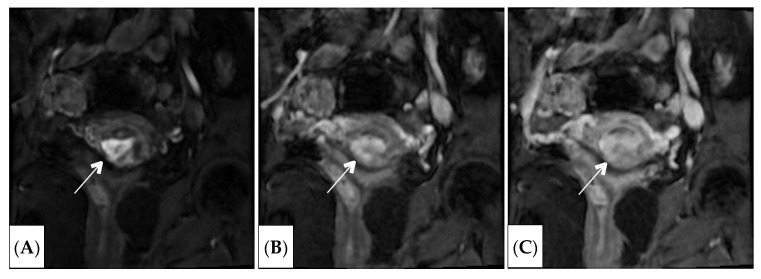
MRI T1WI FS with intravenous bolus contrast (Clariscan 0.2 mL/kg body weight) in the early arterial (**A**), parenchymal (**B**), and delayed (**C**) phases, revealing early hypervascular enhancement of the chorion (indicated by the arrow) and pronounced thinning of the underlying uterine wall.

Given the suspicion of an ectopic pregnancy in the caesarean section scar, the patient underwent examination of magnetic resonance imaging. The study was conducted using a GE magnetic resonance imaging scanner, Signa Architect 3.0 T (GE Healthcare, Anaheim, CA, USA), in T2WI, DWI, T1WI, and T1WI FS modes in three planes (axial, coronal, sagittal) (Figure 1 and Figure 2).

As a result of the MRI study, the uterus was in the anteflexion position, enlarged in volume, corresponding to the gestational age (the uterine dimensions were longitudinal diameter about 7.5 cm, transverse 8.6 cm, anteroposterior 6.6 cm). The uterine walls were of uneven thickness. The cervix was up to 3.3 cm in length. The external and internal os were narrow and closed. A scar niche from the cervix was noted, with prolapse into the area of the internal os. The chorion thickness was up to 1.7 cm. The chorion exhibited a heterogeneous MR signal. A gestational sac was detected in the uterine cavity with one fetus in a longitudinal position. The chorion was annular and located circumferentially along the uterine walls, with a tendency to be positioned in the area of the uterine isthmus.

A cesarean section scar was visible in the lower uterine segment, with a niche resembling a hernia-like protrusion. It was located centrally and laterally on the right. A fragment of the chorion was present in the scar niche and adjacent to the internal os. Given the thinning of the uterine wall, pathological invasion into the area of the thinned scar could not be ruled out. The thickness of the cesarean section scar was up to 0.15 cm of invasion, so the patient underwent organ-preserving surgery.

As preparation for surgery, uterine artery embolization was performed. Under local anesthesia with lidocaine solution, the radial artery was punctured and a introducer sheath was inserted. The left and right uterine arteries were sequentially catheterized. Arterial embolization was performed using Bearing 710–1000 µm material (Merit Medical System, South Jordan, UT, USA). The contrast agent was Omnipaque 350 (Nicomed, Hatboro, PA, USA), 200 mL. Control angiograms showed no contrast enhancement of the pathological arterial plexus.

As preparation for surgery, uterine artery embolization was performed. Under local anesthesia with lidocaine solution, the radial artery was punctured and a introducer sheath was inserted. The left and right uterine arteries were sequentially catheterized. Arterial embolization was performed using Bearing 710–1000 µm material. The contrast agent was Omnipaque 350, 200 mL. Control angiograms showed no contrast enhancement of the pathological arterial plexus. The second stage, performed on the same day, included laparoscopy, adhesion separation, vacuum aspiration of the gestational sac, uterine cavity curettage, hysteroresectoscopy, and coagulation of the gestational sac bed.

Vacuum aspiration of the gestational sac was conducted. Curettage of the uterine cavity was performed. In the area of the upper third of the cervical canal with the transition to the uterine isthmus, the post-cesarean section scar was detected along the anterior wall. This scar formed an irregularly shaped “niche” with dimensions of 10 mm wide, 4 mm long, and up to 8 mm deep, with uneven walls. In the area of the niche extending to the cervix, the gestational sac and the annular chorion were visible, located along the posterior, lateral, and partially anterior walls of the cervical canal. This confirmed the correctness of the MRI diagnosis. Hysteroresectoscopy was performed. The remnants of the chorion were removed with a U-shaped electrode, and the gestational sac bed was coagulated with a ball electrode. A control hysteroresectoscopy revealed a clean uterine cavity with smooth walls. The laparoscopy was admitted. Metroplasty with excision of the thinned fragment was performed. The scraping of the uterine cavity was sent for histological examination.

According to the morphological examination data, chorionic villi from early gestation were present. There was scattered infiltration by neutrophilic leukocytes and lymphocytes. Zones of gravid mucosa of the uterine body were also identified. Visible areas of the myometrium were partially replaced by fibrinoid, some of which were embedded in fibrin masses. Neutrophilic infiltration was likely associated with inflammation in the uterine scar, which contributed to inadequate healing and its overstretching and thinning.

To elucidate the pathogenetic aspects of pathological chorionic invasion, an immunohistochemical study was performed to assess the number of decidual cells, smooth muscle cells, and trophoblast invasion.

**Figure 3:**
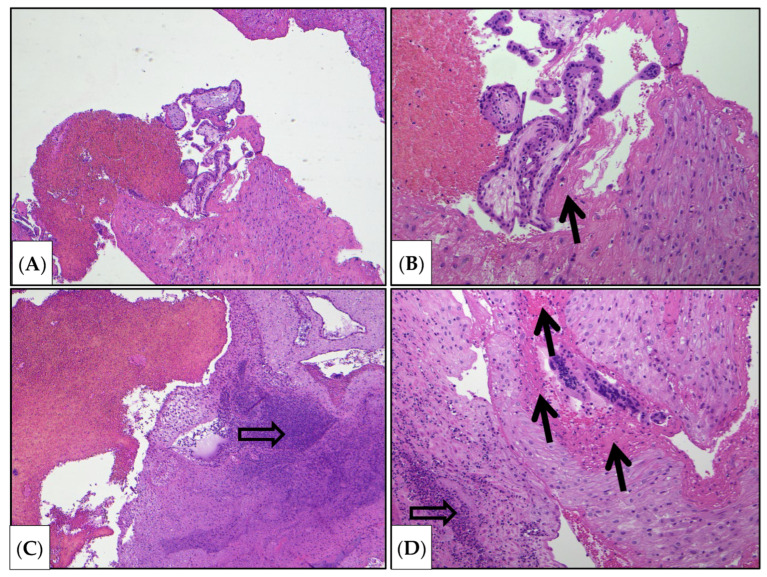
Histological scraping examination (hematoxylin and eosin staining). Chorionic villi, fragments of decidual tissue, areas of myometrium, and blood were identified (**A**,**B**), fibrinoid (protein coagulate), presented as a homogeneously eosinophilic substance (marked with a black arrow). The depth of invasion could not be determined due to tissue fragmentation. However, areas lacking decidual cells with pronounced neutrophil infiltration were present at the suspected sites of invasion (**C**), indirectly indicating pre-existing inflammation in the scar area. The sites of invasion were accompanied by eosinophilic homogeneous masses consisting of coagulated plasma proteins (**D**) (marked with black arrows) (fibrinoid); (marked with hollow arrows) (Inflammatory infiltration (neutrophils predominate) was noted). (**A**) ×40, (**B**) ×100, (**C**) ×40, (**D**) ×100.

**Figure 4:**
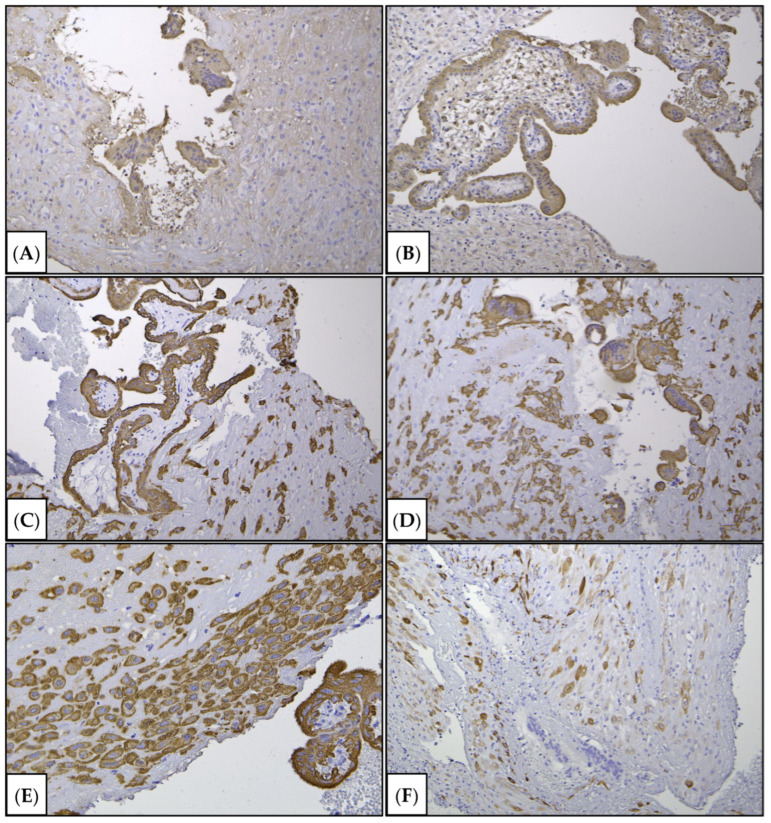
Immunohistochemical study. (**A**,**B**) Staining with primary antibodies to human chorionic gonadotropin beta; (**C**,**E**) with primary antibodies to cytokeratin 8. Chorionic villi were intensely stained, indicating the functional integrity of the trophoblast. Cytokeratin 8 stains villous and extravillous trophoblasts. (**F**) Staining with primary antibodies to desmin (a marker for smooth muscle cell and small decidual cell marker), (**A**–**D**) ×100, (**E**) ×200, (**F**) ×200.

**Figure 5:**
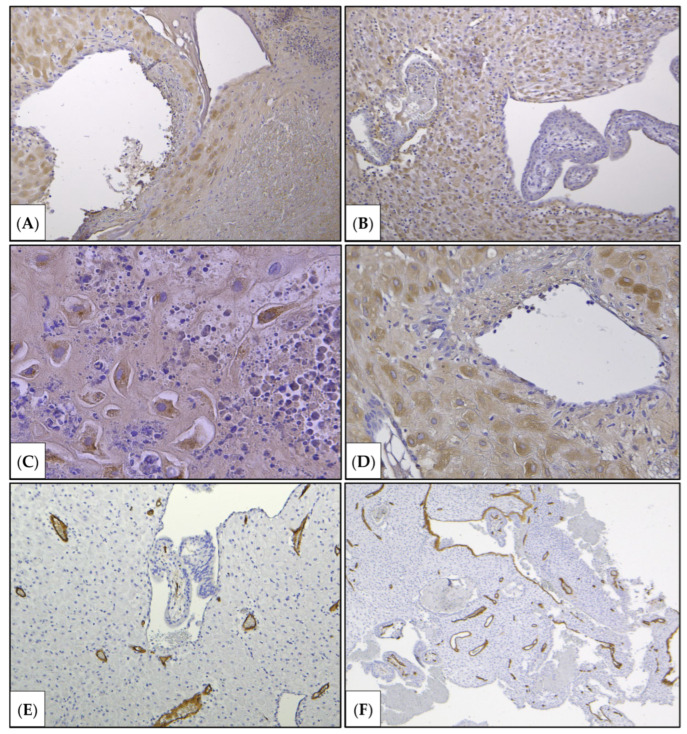
Immunohistochemical study. (**A**–**D**) Immunohistochemical staining with primary antibodies to IGFBP-1 (a decidual cell marker). Inflammatory infiltration of the decidual plate, represented by neutrophils (**C**); lymphocytes. There were decidual with signs of lesions. Decidual cells within (**A**–**C**) and outside (**D**) the area of invasion. (**E**,**F**) Staining with primary antibodies to CD34 (an endothelial cell). Blood vessels of the chorionic villi (**E**) and decidua (**D**,**F**); areas of damage to the decidua and myometrium, inflammatory infiltration, localized disappearance of decidual cells; (**A**) ×100, (**B**) ×100, (**C**) ×400, (**D**) ×200, (**E**,**F**) ×40.

Decidual cells were not observed in the areas of suspected invasion (Figure 3, Figure 4 and Figure 5).

### 2.1. Histological Study

Sections 4 µm thick were cut from paraffin-embedded blocks using the rotary microtome Accu-Cut SRM200 (Sakura, Tokyo, Japan). Microsections placed on glass slides were deparaffinized and rehydrated through a graded ethanol series, then washed in water and stained with hematoxylin and eosin (LLC. “BioVitrum”, Moscow, Russia, article: 07-006) or the Mallory trichrome kit (LLC. “BioVitrum”, Moscow, Russia; #21-036,) for detection of connective tissue according to Mallory (LLC. “BioVitrum”, Moscow, Russia; article: 21-036). The sections were then dehydrated and mounted in Vitrogel (LLC. “BioVitrum”, Moscow, Russia; article 12-005) for further microscopic examination.

### 2.2. Immunohistochemical Examination

Sections from the paraffin blocks were mounted on lysine-coated glass slides (Menzel-Glaser Polysine, Braunschweig, Germany), rehydrated, and subjected to heat-mediated antigen retrieval in citrate solution (pH 6.0). Sections were blocked for one hour at room temperature in 10% goat serum and 0.1% Tween-20 in Tris-buffered saline (TBS), then incubated overnight at 4 °C with primary antibodies specific to the antigen on the section. Slides were washed with phosphate buffer. The interaction of primary antibodies with the antigen was visualized using a horseradish peroxidase conjugate specifically bound to secondary anti-species antibodies. PrimeVision (antibodies to mouse/rabbit IgG-HRP/DAB) (LLC. “Primebiomed”, Skolkovo, Russia, article N:78-310004-55) was used to detect bound primary antibodies, followed by counterstaining with Mayer’s hematoxylin solution (LLC. “BioVitrum”, article: 05-002/S). Dehydration was performed in a graded ethanol series, followed by mounting with Vitrogel (LLC. “BioVitrum”, article 12-005). The reaction resulted in the development of a brown chromogenic substrate. An aqueous solution of 3,3-diaminobenzidine tetrahydrochloride (DAB) was used to stain the immunohistochemical reaction product. Positive immunohistochemical reaction products were identified by brown staining of the membrane and/or cytoplasm of cells. For the negative control, sections underwent standard immunohistochemical procedures without incubation with primary antibodies.

The positive control was selected according to the manufacturer’s specifications. For specimens, we used mouse antibody (mAb) to cytokeratin 8 (Clone CK-8-IS1-RTU; Novocastra, Apeldoorn, The Netherlands) and to human chorionic gonadotropin (HCG) (clone ZSH 17-RTU; NordiQS, Shanghai, China) for cytotrophoblast and syncytiotrophoblast detection; clone GMOO76-RTU (LLC. «Primebiomed», Moscow, Russia) as a marker of fibroblasts, myofibroblasts, and small decidual cells; mouse monoclonal antibodies to IGFBP-1 (clone EPR12315, ab170911, Abcam, Shanghai, China) as a specific marker of decidual cells; and the endothelial marker CD34 (clone QBEnd/10-RTU; Shanghai, China). Specimen microscopy was performed using a Leica microscope system, consisting of the Leica DM2500 microscope and Leica DFC290 video camera (Leica, Wetzlar, Germany). The reaction products appeared as a brown color.

According to the morphological examination data, chorionic villi from early gestation were present, and edema of the villous stroma, some of which were embedded in fibrin masses. Fragments of decidual tissue showed circulatory disturbances and scattered infiltration by neutrophilic leukocytes and lymphocytes. Zones of gravid mucosa of the uterine body were also identified. Decidual cells were not observed in the areas of suspected invasion (Figure 3, Figure 4 and Figure 5). Given the type of surgery and the fragmentation in the scraping, it was not possible to determine the depth of chorionic invasion.

The study was approved by the Ethics Committee of FSBI “National Medical Research Center for Obstetrics, Gynecology and Perinatology named after Academician V.I. Kulakov” Ministry of Health of the Russian Federation №4 7 February 2022. Informed consent was obtained from the subject involved in the study. The patient signed informed consent for the publication of their data and images.

## 3. Results and Discussion

This case highlights the challenges of diagnosing pregnancy in a cesarean section scar. Ultrasound made it difficult to assess the location of the gestational sac and the presence of chorionic invasion of the uterine wall. But, ultrasound enabled identification of the cause of the spotting and raised early suspicion of pregnancy in the scar.

The final diagnostic method was magnetic resonance imaging, which allowed evaluation of the gestational sac (GS) localization. It was found that the GS was located in the uterine cavity, but specifically in the lower part, within the scar niche at the uterine isthmus. This variant could be classified as type II ectopic pregnancy in the scar [11]. Depending on the gestational age, different surgical options are possible for PAS [17,18,19,20,21,22,23,24,25].

In addition, MRI examination revealed signs of chorionic invasion in the scar and uterine isthmus, as well as significant thinning and deformation of the scar zone. Furthermore, the gestational sac location in the highly vascularized area of the uterine isthmus indicates a high risk of massive bleeding [17].

Determining the type of vascularization is an important task for assessing treatment strategy and tactics. Thus, we have previously shown that different types of vascularization can occur in PAS [26].

Several scientific studies have examined the assessment of cesarean section scars for pregnancy planning, which may be applied to this clinical case. MRI measures the scar thickness, which should normally be at least 3 mm, as well as the parameters of the scar niche, including the depth, width, and longitudinal dimension of the “niche”. The thickness of the intact myometrium above the scar is also measured [27].

Currently, there are various treatment strategies for ectopic pregnancy: medication, endoscopic, and surgical methods, but there is no single, generally accepted algorithm of action [28].

Some foreign scientists point out the high effectiveness of drug treatment for ectopic pregnancy in the early stages of pregnancy, while in later stages there is an increased risk of bleeding and more time is required for the pregnancy to cease developing [18,19,20].

Other authors believe that surgical intervention leads to the most rapid decrease in β-hCG concentrations and termination of pregnancy, while in cases of non-viable pregnancy, it is proposed to administer methotrexate both locally and systemically intravenously [21].

However, it is worth noting that after conservative treatment, pregnancy occurred in only 50% of patients, which is not a high rate [23].

To prevent bleeding during surgery, some authors recommend temporary bilateral ligation of the uterine arteries during laparoscopy in combination with hysteroscopy, while other clinics perform uterine artery embolization followed by curettage of the uterine cavity [24,29,30].

According to the 2024 Russian Federation clinical guidelines, surgical and medical (methotrexate) treatment are recommended for all forms of ectopic pregnancy [31]. However, it is unknown whether methotrexate is the drug of choice.

There is also the Lieto’s triangle (trigonum vesicae), which is located in the lower part of the bladder and is bounded by the orifices of the ureters and the internal opening of the urethra. This triangle has a smooth mucous membrane and differs in structure from the rest of the bladder. The most severe placental invasion occurs in the area of the vesical triangle (triangle vesicouterinae).

One of the most severe variants is placental villi invasion in this area of the triangle. Blood supply above the vesical triangle comes from the superior vesical, superior vaginal, and uterine arteries, while below it, the blood supply involves more complex anastomoses. In this clinical case, the chorion was located and invaded in the projection of this triangle. Central chorion previa was considered to carry a high risk of obstetric hemorrhage. Although the scar thickness from caesarean section was 0.15 cm, there were no signs of dehiscence. Histological examination did not allow assessment of the depth of invasion due to surgical intervention and fragmentation of the curettage material. Histological examination revealed inflammation in this area (neutrophils and leukocytes), local necrosis of the uterine wall, and an increase in fibrinoid deposits, which in our view was highly specific for abnormal placental invasion (Figure 1).

Immunohistochemical examination revealed a localized absence of decidual cells (confirmed by the absence of IGFBP-1 staining). There were no decidual cells in the niche area (confirmed negative staining for IGFBP-1), which is a specific sign of invasion [32,33]. The trophoblast was intensely stained with cytokeratin 8 and beta-hCG, demonstrating the persistence of its invasive properties.

Thus, this diagnostic method (using contrast during MRI) and the surgical removal of the gestational sac were chosen because the woman did not plan to have more children and did not wish to continue the pregnancy.

Therefore, we could demonstrate both contrast-enhanced MRI, which cannot be demonstrated in such cases, as well as histological and immunohistochemical studies.

Gadolinium-based contrast agents are commonly used in magnetic resonance imaging (MRI) to enhance visualization and characterization, including the vascularity of the lesion. MRI is generally considered a safe procedure during pregnancy; however, concerns have been raised regarding the safety of gadolinium-containing contrast agents, particularly in the first trimester. Limited studies have been conducted to evaluate the safety of gadolinium-containing contrast agents in pregnant women, and the results have been inconsistent. Therefore, the safety of intravenous contrast administration during pregnancy, particularly in the first trimester, remains questionable. Larger, long-term studies are needed to clarify the safety of gadolinium-containing contrast agents in pregnant women and their potential effects on the fetus and neonate. Until conclusive evidence is available, healthcare professionals should carefully weigh the benefits and risks of using gadolinium-containing contrast agents during pregnancy and, if necessary, consider alternative imaging modalities, such as non-contrast MRI or ultrasound [31].

Many clinical conditions require radiological diagnostic exams based on the emission of different kinds of energy and the use of contrast agents, such as MRI, ultrasound examination, and others. Pregnant patients who should be submitted for diagnostic examinations with contrast agents represent a group of patients with whom it is necessary to consider both maternal and fetal effects [16].

In this case, the patient, considering the potential health risks and personal preferences, decided to terminate the pregnancy and plan another pregnancy in the near future. This allowed us to conduct an MRI study with intravenous bolus contrast, which was particularly important for assessing the vascularity of the uterine wall and chorion, as the patient was scheduled for uterine artery embolization. We were also able to conduct a post-termination pathological examination, which enhances the uniqueness and significance of this case report.

## 4. Conclusions

Thus, in our case, the cervical pregnancy lasted 8 weeks and 5 days, progressing and developing. MRI is the imaging method of choice in complex cases of cervical pregnancy. The cesarean section scar showed signs of failure, including thinning, the formation of a scar niche, and signs of inflammation. To minimize the risk of blood loss, uterine artery embolization was performed, which is the most modern and effective method of preparing for surgery. In the most complex cases, the use of contrast can provide information about the location of the gestational sac, the depth of invasion, and the need for continuation or termination of the pregnancy. The pregnancy was undesirable for the patient, so we had the unique opportunity to test the diagnostic method, perform intravenous bolus contrast during MRI, and obtain histological specimens.

The gynecologists were able to remove the chorion and preserve the uterus while simultaneously performing metroplasty, allowing the patient to plan future pregnancies.

## Data Availability

The original contributions presented in this study are included in the article. Further inquiries can be directed to the corresponding author.

## Abbreviations

The following abbreviations are used in this manuscript:

CS	Cesarean section
GS	Gestational sac GS
PAS	Placenta accreta spectrum disorder
SNP	Scar niche pregnancy
